# Report of the Committee Appointed by the American Medical College Association to Consider and Recommend a Plan of Registration of Medical Colleges in This Country

**Published:** 1880-08

**Authors:** N. S. Davis, S. D. Gross


REPORT OF THE COMMITTEE. Appointed by the American Medical College Association to Consider and Recommend a Plan of Registration of Medical Colleges in this Country. Read May 31, 1880.

At the annual meeting of this Association, held in Buffalo, N. Y., June, 1878, a communication was received from a foreign source asking for a list of American medical colleges in good standing, the degrees from which should be recognized by the medical institutions of other countries. Questions of a similar character had been asked many times before, both at home and abroad.

Owing to the number and varied character of the medical schools in this country, there was some difficulty or embarrassment in returning a proper answer; and after some discussion a committee of three was appointed to consider and recommend a *plan* for the registration of American medical colleges.

A report of considerable length was prepared and submitted to the regular meeting of the Association in Atlanta, Georgia, May, 1879. Some of the more important recommendations appended to that report were adopted, but the report, aside from such recommendations, was re-committed or referred back to the same committee with instructions to revise and condense the same and report it to the next annual meeting. In accordance with these instructions the report has been carefully revised, or, more properly, re-written, and is now respectfully presented by the undersigned as follows:

In devising a plan for the registration of medical colleges, which shall designate those whose diplomas should be recognized or respected at home and abroad, the question immediately arises, What shall be the standard by which such institutions shall be judged? In considering what constitutes a proper standard for a college, we must determine: First, What constitutes a proper or adequate medical education? Second, What is the minimum length of time required for a fair acquisition of such an education? Third, What part of such time should be spent in direct attendance on a medical college? and Fourth, What are the appliances, means of illustration, and facilities for imparting practical knowledge, that are necessary to constitute a college capable of performing all the functions required of such an institution?

The first of these questions may be answered by saying that an adequate medical education is such as gives to the student a fair practical knowledge of all branches of medical science, and a mental discipline sufficient for the proper use of such knowledge in the practice of the medical art. We use the words "practical knowledge" as implying something more than mere verbal knowledge. For instance, a student might study a text-book on human anatomy until he could describe every bone in the skeleton, state the origin and insertion of every muscle, and give the distribution of the blood-vessels and nerves, and thereby sustain a fair verbal examination in that department. Yet, if he had never either dissected a subject himself, or witnessed the actual demonstration of each part by a competent teacher, he would be entirely devoid of that *practical* knowledge necessary to guide him in the operations of surgery. The same is true of most of the branches of medical science. Therefore, a practical knowledge of these various branches necessitates demonstrative teaching and personal manipulation, which can be provided in an adequate degree in medical colleges only. The amount of medical knowledge requiring for its proper acquisition this kind of teaching, makes it necessary that at least one-half of the whole time allotted as the period of medical pupilage should be spent in the medical college.

As there are none at the present time who seriously claim that less than *three* full years should be devoted to a diligent study of medicine, before graduation or commencement of practice, we may answer the second and third questions by stating that the minimum length of time required for gaining an adequate knowledge of medicine should be not less than three years; and that at least one-half of each of these years should be spent in a proper medical college. And this makes it necessary that the medical colleges, to be capable of performing their functions properly, should extend their annual term of active and obligatory instruction to six months of each year. It is obvious, also, that in answering the fourth question, we must insist that the very objects for which medical colleges are needed make it necessary that each one should not only provide a full curriculum of studies, a full corps of teachers, and an annual term of six month's instruction, but also a sufficient supply of anatomical material for demonstrations and practical dissections; a chemical apparatus and laboratory for practical or analytical chemical work; a histological and physiological laboratory for practical and microscopic work; a museum containing a collection of specimens of normal and pathological anatomy sufficient for illustrating the departments of pathology, practical medicine and surgery; and access for the students to a hospital containing a daily average of not less than thirty patients actually in the wards for treatment and accessible for clinical instruction. A college which is so organized, or so located, that it does not or cannot provide for the daily use of its students all the resources here named, both for didactic and practical instruction, is not capable of performing all the functions that should be performed by every such institution that receives the patronage of the profession and the public. So long as the medical college diploma is recognized as a sufficient license to practice, every college asking for recognition should be required, in addition to possessing the foregoing means for adequate instruction in all departments, to exact proper evidence from every student that he possesses at least a good English education before allowing him to become a matriculate of the college; and after matriculation he should be required to attend *three* regular annual terms of medical college instruction of not less than six months each, and the last of which should be in the institution granting him a diploma.

Such are the views of your committee in regard to the *standard*, to which all medical colleges should be required to attain, before their diplomas should be regarded worthy of universal recognition.

And if this College Association should adopt and enforce two amendments to its articles of confederation proposed at the last annual meeting, and now lying on the table awaiting final action at this meeting, it would speedily bring a large majority of the colleges located within reach of hospitals accessible for adequate clinical instruction directly up to the standard proposed. One of these amendments provides for the enforcement of a proper standard of preliminary education before admitting the student into the college, and the other for demanding attendance on *three* regular annual courses of medical college instruction before graduation. It is greatly to be desired that both these amendments shall be adopted unanimously by this Association at its present session. Until such action is taken, however, your committee have no alternative but to adopt the standard presented by the present articles of confederation of this Association as a basis or guide for the registration of American medical colleges. By a careful examination of the annual announcements of the colleges of legitimate medicine in the United States for 1879-80, we find about three-fourths of the whole number complying fully with all the requirements for membership in this Association. We find several of the remaining ones complying with all the regulations in regard to time, means, and amount of instruction furnished and qualifications required of the student, but failing in some particulars in relation to the exaction of *fees* for instruction. And a few come short more or less, either in the amount of instruction furnished, or in the time of attendance required, or both. Some of these serious shortcomings are in institutions where we should least suspect their existence. For instance, in the Medical School of Harvard, at Boston, there is certainly nothing in their annual catalogues to hinder a student from receiving the degree of M. D. from that institution by an attendance on simply one college year, which means merely nine months. The language of their catalogue for 1879–80, touching this point is, under the head of *Division of Studies*, as follows: "Students may be admitted to advanced standing in the regular course; but all who apply for admission into the second or third class must pass an examination at the beginning of the year in the branches already pursued by the class to which they seek admission, and furnish a satisfactory certificate of *time* spent in medical studies." Again, under the head of *Requirements for a Degree*, we were told that "Every candidate must be twenty-one years of age, and of good moral character; must give evidence of having studied medicine three full years; have spent at least one continuous year at this school; have presented a satisfactory thesis; and have passed the required examinations."

In turning to the examinations required in each year, we find those of the first year to be Anatomy, Physiology, and General Chemistry. Those for the second year, Medical Chemistry, Materia Medica, and Pathological Anatomy. Now, it strikes us that he must be rather a dull young man who cannot take the necessary books, get some regular practitioner to lend the use of his name as a preceptor, and in two years make himself sufficiently familiar with the language of his text-books, that he can pass a fair examination, either oral or written, in Anatomy, Physiology, and General Chemistry, in connection with at least two out of the following three: Medical Chemistry, Materia Medica, and Pathological Anatomy. And if he can do this he can step directly into the third or graduating class, and complete his course by attending *one* college year, without ever having set foot inside of any other medical school. We are sorry to perceive that in regard to permitting students to take advanced standing in such a way as make the degree accessible by attendance on only one collegiate year of medical instruction, the example of Harvard has been followed by the Medical Department of Yale, and possibly some others. If we have not misinterpreted the catalogues, the very school which has made more pretentious appeals for the sympathy and support of the whole profession, on account of the great advancements she is making in elevating the standard of medical education in this country, is to-day offering her diploma for a less amount of medical college attendance and instruction than any other respectable school in this country.

Indeed, in reviewing the annual catalogues and announcements of the medical colleges generally, nothing has struck us so forcibly as the vast amount of medical instruction provided by these institutions, and to all of which the students are most cordially invited, to some part of it without money and without price; compared with the very modest amount they are actually required to take.

The almost stereotyped flourish is something like the following: "The preliminary course of lectures in this institution will commence on the 15th of September and continue until the opening of the regular college term. Three lectures per day will be given on very important topics, by members of the college faculty, besides the usual clinics in the hospitals and dispensaries. This course is free to all students. The regular winter term commences on the first Monday in October, and continues until the last Tuesday in February, when the public commencement exercises will be held and degrees conferred upon the members of the graduating class. The regular spring and summer course of instruction will commence on the first Monday in March, and continue three months. This is a very important course for the student to attend, as many topics of great importance will be taught for the consideration of which there was not sufficient time in the regular winter term. "This course is often free to all regular matriculates of the college, at other times a fee of $20, $30 or $50 is charged, the same to be credited on the fees of the next winter term." It is thus seen that instruction in this institution continues through nine months of the year. But attendance on the regular winter term *only* is *obligatory* upon the student, that on the spring course being entirely optional and not counting as a course in the requirements for graduation." This is a very moderate representation, of the general average of pretention. Nine months of instruction, all of which is alleged to be very important, and yet only five months, or just a trifle more than one-half of the whole, is the student actually required to pay any attention to. Again, look at the self-stultification presented in the claim that all the instruction provided is important and much of it on topics that could not be included in the regular winter term for want of time. If this claim is true, common sense would dictate either the extension of the winter term or the making of attendance on the spring term obligatory.

Still another phase of these shams consists in cutting up the general departments of medicine, surgery and obstetrics, by carving out of them something less than a dozen *specialties* with a lecturer or full professor in each one. This swells the list of members of the faculty and instructors to formidable proportions, and helps to back up the general claim of great advancement in the facilities for instruction. But when we turn to the time allotted to each of these additional teachers in which to give their highly important amount of instruction, we find the length of the term *obligatory* upon the student substantially the same as before, and the number of hours daily devoted to instruction also the same.

Nothing is more plain, therefore, than the fact that all this array of special additional teaching either takes the place of a similar amount of teaching under the general heads, or is crowded into the terms, attendance on which is entirely optional, namely, the preliminary course and the spring and summer terms—the preface and the supplement; or, to express the actual facts more exactly, these additional special branches come in to occupy a part of the time of the regular obligatory college term, and by shortening just so much the time allotted in said term to the general branches, a corresponding amount of instruction in these goes over to help make up the purely voluntary spring and summer course, when, as a general rule, three-fourths of the students have returned to their homes or their preceptor's offices. To cap the climax in this complicated effort to show a great advance, and yet, so far as real exactions upon the students are concerned, stand quite still, many of the colleges do not even require the students to be examined in any but the seven main branches of medicine, viz.: anatomy, physology, chemistry, materia medica, practical medicine, surgery and obstetrics. It does not require a very rigid annalysis of the facts here alluded to, or many years of practical experience in connection with medical colleges, to see very clearly that all this array of optional medical instruction has served to diminish the completeness and harmony, without increasing the amount of the real medical knowledge communicated to the great body of young men attending the colleges. For no fact is better known to the members of this Association than that three-fourths of the whole number of young men who study medicine actually *attend only* just so much medical college instruction as is made obligatory upon them for the attainment of a diploma. Consequently, the college that has during its obligatory winter term of five months, or twenty weeks, 150 students, will find from 25 to 40 remaining to enter upon the voluntary spring term commencing early in March. By the end of April, half of these have disappeared, one by one; and by the end of the term it will be doing well if a baker's dozen be present at the closing lecture. If the college be in one of the great commercial cities and has a class of 400 or 500 students attending its obligatory term, 100 to 150 may enter their names for attendance on the voluntary spring term, but as the term progresses, the same dwindling takes place as in the other case. With some the pocket money gets low; with others, it is becoming too warm; still others find they are needed at home to aid their preceptors or somebody else; and the faculty of instructors have cause for special congratulation if they have from 50 to 75 actually in the lecture rooms at the close of the term. Now how much better it would be for all parties concerned if the regular obligatory term was extended to six months, whereby one month more of systematic instruction in all departments were given to the whole 400 or 500 students. instead of two months additional voluntary instruction to a number ranging from 50 to 150. The effects here seen in one college apply equally to all the colleges and to the whole mass of medical students. The present state of things has grown out of the efforts of the colleges to yield to the constantly increasing demands of the profession and of the people for a higher standard of attainment on the part of medical men. Knowing that the controlling motive of the student is to obtain the college diploma with as little expenditure of time and money as possible, and influenced, whether consciously or not, by strong feelings of mutual jealousy and active rivalry, every device has been resorted to for increasing the show of instruction and advancement without increasing materially the obligatory demands upon the time and attainments of the student before he gets his degree.

Hence it is that we are in the midst of an era of an enormous show of medical instruction, as represented in the college announcements, a trifle more than half of which the students are obliged to attend, while the other half goes mainly to empty benches. The latest addition to this side-show business is the notice sent out by the faculty of Harvard, for which she is now receiving a fresh supply of gratuitous advertising in the medical journals, stating that hereafter she will add a fourth year of medical instruction to her already extended system. True the notice states that it will be mainly a continuation of the studies of the third year, and that attendance upon it will be entirely optional with the student. But if the latter does graciously condescend to attend this fourth year before he graduates, he is permitted to have added to his title of Doctor of Medicine, those remarkable words, "*cum laude.*" We had always supposed that any student who desired had the privilege of adding a fourth, fifth or even sixth year to his medical studies in any medical college in the country. At any rate we have known several rather dull young men who were obliged to attend a fourth year, and one even the fifth year before obtaining a degree. But this era of make-shifts and pretentions, of more show than substance, of superabundant provision for instruction without requiring the student to attend more than half of it, must soon come to an end. It is in the nature of things only a transition period. This College Association must perpetuate its existence, and move steadily on to the perfection of its work by adopting the pending amendments in relation to preliminary education and the exaction of attendance upon three annual courses of college instruction before graduation; and in all other important particulars combining and harmonizing the colleges, and perfecting the details of a fair and honorable system of medical education; or, failing in this, the profession and the people will unite on such legislation as will establish independent boards of examiners in every State, and take the matter of licensing candidates to practice medicine entirely out of the hands of the colleges, thereby leaving them to to compete with each other solely as institutions for imparting instruction.

Already the drift of popular demand is plainly in the latter direction. In making this report, we have omitted a detailed list of colleges that comply with the requirments of this Associations, as well as of those that do not comply, simply because that special duty was, at the last meeting, assigned to a regular standing committee, and we thought a duplication of the same work unnecessary. Respectfully submitted,

N. S. DAVIS,
S. D. GROSS.

